# Food and Teeth

**Published:** 1918-10

**Authors:** 


### FOOD AND TEETH

That the nature of the staple foodstuffs of a country have at least some determining influence upon the healthy development of the teeth and jaws of its inhabitants is one of the few tenets of belief upon which all dentists and odontologists are in agreement. The dental apparatus must be exercised, and the functional exercise must be of the right kind. And it is readily conceivable that there might be over-exercise as well as under-exercise of the jaws, and also that the habitual use of certain foods might evoke a more-or-less perverted muscular action or a wrongful and bone-deforming interplay of the muscular forces. A recognized authority may lay it down that this or that particular dietary is best for the teeth; but evidence rather than authority is what is asked for nowadays, and an inquirer would feel that he was moving towards firmer ground if he could but get to know whether there was any *constant* and fairly-definite physical character in the staple foods used by the various peoples who had fine teeth. As relevant evidence about the foods used in times long past is unprocurable, chief attention is given to acquiring information about the foods and eating habits of present-day living people, both primitive and civilized. That an inquiry of this sort is not so simple as it might appear is made apparent by the conflicting nature of the testimony of different observers. And the value of such odds and ends of evidence as are at present available has eventually to be discounted and checked against a certain unregarded assumption that is commonly made—*i. e.*, that while the foodstuffs of many civilized peoples have altered considerably, those of aboriginals or savages have remained unchanged. As Dr. Stanley Colyer pointed out in the *Dental Record* (pp. 1 and 629, 1916), the dietaries, even of aborigines habitated far from the regular trade routes, become subject to changes that may affect the teeth and undeniably sometimes does play havoc with their physical and moral well-being.

Nearer home, too, in the case of the potato-fed Irishman and the porridge-fed Scot, we have to remember that the Irish people were physically and dentally sound *before* the advent of the potato, and that the Scot did not always, as nowadays, consume his oats mainly in the form of soft porridge.

From two fairly-recent travel books, one dealing with Siberia and the other with the north-west Amazon region, the following scraps of information have been gleaned. M. A. Czaplicka states in "My Siberian Lear" (Mills & Boon), that "while the natives of the north live mainly on fish and meat, the Steppe people have a more varied diet. Meat forms about one-third of the whole quantity of provisions they consume." * * * "The Tartars also eat a large quantity of the roots of two plants called *kandik* and *sarana*." Speaking of the Siberiake, the author refers to the "incessant chewing of *syera* (larch rosin), and the perennial champing of the seeds of Siberian cedar cones."

In "The North-West Amazons" (Constable: London, 1915), by Captain Thomas Whiffen, there is an interesting chapter on "the food quest." The staple food of the Amazonian native is the manioc (*Manihot utilissima*). The manioc roots or tubers undergo at the hands of the "untutored" native a quite elaborate process, the chief objects of which are to eliminate the poisonous qualities and prepare the flour from which the cavassa bread is made. "Cavassa bread is leathery and tough." A great variety of animals are eaten by the Amazons; monkey is "the most ordinary food of the Indian," and monkey flesh is "usually tough and invariably insipid." "Of birds, parrots are the most plentiful, and the toughest. For a hard, tasteless, and unappetizing meal, commend me to the carcass of that noisy bird." And again, "Macaw, curassow, *piuri* and *panji*, mocking bird, toucan, and egrets all go to the family pepper-pot of the successful hunter, with the turkey of these parts, pigeons, partridges, herons, ducks, and geese." Frogs, snakes and reptiles are considered delicacies. The teeth of these natives are briefly described as "big and even, and very rarely found projecting."

The above extracts are here quoted merely as the statements of recent travellers who had some scientific training, and they are not at all offered in refutation of the remarkable proposition advanced by Major H. P. Pickerill in last month's issue of the *Dental Record.* Indeed, an enormous amount of work has yet to be done, in the gathering of data alone, before any one can be in the position to make very positive or convincing statements on the relation between change of staple foodstuffs and changes in the position of teeth or the shape of jaws.—*Dental Record.*

